# Correlation between artificial intelligence-based IDAScore and embryo morphokinetic parameters: a retrospective cohort study

**DOI:** 10.1186/s12884-026-09662-5

**Published:** 2026-07-17

**Authors:** Lanxiang He, Qiulei Ke, Huizhen Li, Wei Yu Huang, Yiyu Tian, Wen Zhang, Tianzhong Ma

**Affiliations:** 1 Reproductive Medicine Center, Affiliated Hospital of Guangdong Medical University, 57 Renmin Avenue South, Zhanjiang, Guangdong Province 524001 P. R. China; 2 The Second Affiliated Hospital of Guangdong Medical University, Zhanjiang, Guangdong China

## Abstract

**Purpose:**

The rapid advancement of artificial intelligence (AI) has brought new opportunities for embryo evaluation. The integration of AI with time-lapse (TL) imaging technology has significantly enhanced the pregnancy rates in assisted reproductive technologies (ART). However, the fully automated AI-based embryo screening model, IDAScore lacks transparency and specific justifications in assessing the internal processes of embryos, and its interpretability remains limited. The aim of this study is to explore the correlation between IDAScore and embryonic morphological kinetics, providing a convincing explanation for AI-driven embryo assessment and selection.

**Methods:**

A retrospective analysis was performed on clinical data from 102 first fresh embryo transfer cycles involving cleavage-stage embryos, all morphologically graded as 8-cell, grade 1 (8C1), and 210 first frozen-thawed blastocyst transfer cycles with embryos uniformly scored as 4AA, conducted at the Reproductive Medicine Center of the Affiliated Hospital of Guangdong Medical University between August 2023 and March 2025.All embryos were cultured using a time-lapse imaging system and evaluated by the fully automated AI-based scoring model, IDAScore V2.0.Cleavage-stage embryos were stratified into two groups based on their IDAScore: Group A [8cells, IDA:1.0-5.7]n=27and Group B [8cells, IDA:5.8-8.0]n=75. Blastocysts were similarly divided into Group C [IDA:1.0-7.7]n=98 and Group D [IDA:7.8-9.9]n=112. Clinical baseline characteristics, ovarian stimulation parameters, embryonic kinetic data, and pregnancy outcomes were compared across the four groups.

**Results:**

There were no significant differences in baseline clinical characteristics—including patient age, anti-Müllerian hormone (AMH) levels, body mass index (BMI), and basal hormone profiles—among the four groups. In cleavage-stage embryos, Group B (higher IDAScore) showed significantly faster developmental timings compared to Group A (low IDAScore), including time to 3 cells (t3), 4 cells (t4), 5 cells (t5), and 8 cells (t8), as well as the duration from 2-cell to 3-cell (cc2) and from 3-cell to 5-cell (cc3) stages (P < 0.05). Moreover, Group B demonstrated significantly higher rates of high-quality embryos, blastocyst formation, usable blastocysts, and clinical pregnancy compared to Group A (P < 0.05).Logistic regression analysis revealed that the cc2 interval was significantly associated with IDAScore on Day 3 (OR = 0.410; 95% CI: 0.253–0.666; P < 0.001).In blastocyst-stage embryos, Group D (higher IDAScore) exhibited significantly faster development to the 2-cell (t2), 3-cell (t3), 4-cell (t4), and 5-cell (t5) stages, as well as earlier time to full blastocyst expansion (tB), compared to Group C (low IDAScore) (P < 0.05). Additionally, significant differences were observed between Groups C and D in both inner cell mass (ICM) and trophectoderm (TE) grading (P < 0.05). The blastocyst formation rate in Group D was significantly higher, and the miscarriage rate was significantly lower than that in Group C, with both differences being statistically significant. Stepwise logistic regression analysis showed that the time to full blastocyst expansion (tB) (OR=0.780;95%CI: 0.721-0.845;P < 0.001) and trophectoderm (TE) grading (OR=4.224;95%CI:1.633-10.926;P=0.003) remained significant factors influencing blastocyst scoring.

**Conclusion:**

There is a significant correlation between IDAScore and both embryonic morphology and kinetic parameters. Faster-developing embryos are generally associated with higher IDAScore values and demonstrate greater implantation potential. This finding provides a scientific basis and theoretical explanation for the use of IDAScore in embryo selection.

## Background

According to the latest data from the World Health Organization, approximately 17.5% of couples of reproductive age globally may experience infertility [1]. Assisted reproductive technology (ART) remains the most important and effective treatment for infertility, with the ultimate goal of achieving a healthy singleton pregnancy [2]. However, in clinical practice, multiple embryos are often transferred in a single cycle to increase the likelihood of success, which consequently leads to a higher incidence of multiple pregnancies—one of the major adverse outcomes of ART. Multiple gestations are associated with significantly increased risks of neonatal and obstetric complications, including preterm birth, miscarriage, fetal malformations, intrauterine growth restriction, stillbirth, gestational diabetes, hypertensive disorders of pregnancy, and postpartum hemorrhage [3, 4]. Single embryo transfer (SET) is the most effective strategy for reducing the rate of multiple pregnancies in ART. Embryo selection is therefore a critical component of SET. Currently, the most widely used method for embryo assessment in reproductive centers is conventional morphological grading, which involves static observations of embryo morphology at predetermined time points [5]. However, conventional morphological assessment has inherent limitations, including limited informational value, low interobserver consistency, and high subjectivity, which hinder the accurate identification of embryos with high implantation potential [6–8], particularly in younger patients who may have multiple high-quality embryos available for transfer.In recent years, several non-invasive embryo selection techniques have rapidly emerged, such as time-lapse (TL) imaging, metabolomics, and Raman spectroscopy [9–11]. Among these, TL technology enables continuous monitoring of embryo development in vitro, allowing for the detection of abnormal developmental events. This provides additional, dynamic information to support the selection of embryos with higher developmental competence. The temporal dynamics parameters refer to the time required for an embryo to develop to a specific stage starting from fertilization, with fertilization time defined as t0. These parameters include the number of pronuclei, blastocyst morphology, and eight morphological time points: the time of pronuclear disappearance (tPNf), the time of cleavage into 2 cells (t2), and subsequent cleavage into 3 cells (t3), 4 cells (t4), 5 cells (t5), and 8 cells (t8). Additionally, the developmental time from 2 to 3 cells (cc2 = t3 - t2), from 3 to 5 cells (cc3 = t5 - t3), from 3 to 4 cells (s2 = t4 - t3), and from 5 to 8 cells (s3 = t8 - t5) are also considered. Other parameters include the time at which blastocyst formation begins (tSB) and the time of blastocyst expansion (tEB) [12, 13]. TL technology has been shown to effectively improve embryo transfer outcomes and increase clinical pregnancy rates [14, 15].

The application of artificial intelligence (AI) in healthcare is expanding rapidly, with AI being recognized as an efficient tool for diagnostic assistance, treatment evaluation, and prognosis prediction [16]. With its exceptional ability to process large volumes of image and video data, AI has become an ideal tool for analyzing the complex and dynamic morphological variables in time-lapse (TL) videos. The integration of AI and TL technology has paved the way for the automation and objectification of embryo morphology analysis [17, 18]. Research has demonstrated that combining TL with AI to establish embryo selection models can predict embryo quality [19]. The IDAScore model is a fully automated artificial intelligence embryo selection scoring system developed using deep learning and neural networks. This system takes into account the entire process of embryo development and provides real-time, second-level scoring throughout the embryo culture period [20]. However, the internal logic behind the IDAScore scoring algorithm lacks sufficient transparency and interpretability [21]. Previous limited studies have identified correlations between IDAScore values and embryonic morphological developmental progression. However, the independent interpretable embryonic kinetic biomarkers driving these scores reported across distinct investigations remain inconsistent. The iDAScore model was primarily trained on European and American populations. Baseline discrepancies exist between European and American women and Chinese women of childbearing age in terms of basal AMH levels, follicular development, and embryonic cleavage timings. Direct application of this model to Asian populations may introduce systematic bias; for instance, embryos from Chinese patients tend to be under- or over-scored even under identical cleavage rates. Existing studies lack external validation based on Chinese reproductive-age populations. Consequently, clinicians and patients in China remain skeptical about embryos evaluated and screened by the iDAScore model. Additionally, the “black-box” limitation of this algorithm has not been adequately addressed. Therefore, further research focusing on specific embryonic morphokinetic biomarkers underlying embryo assessment and selection is warranted. Such work can provide robust clinical evidence to support clinical decision-making and broader application of the IDAScore model, thereby improving confidence among both clinical practitioners and patients.

## Materials and methods

**Fig. 1:**
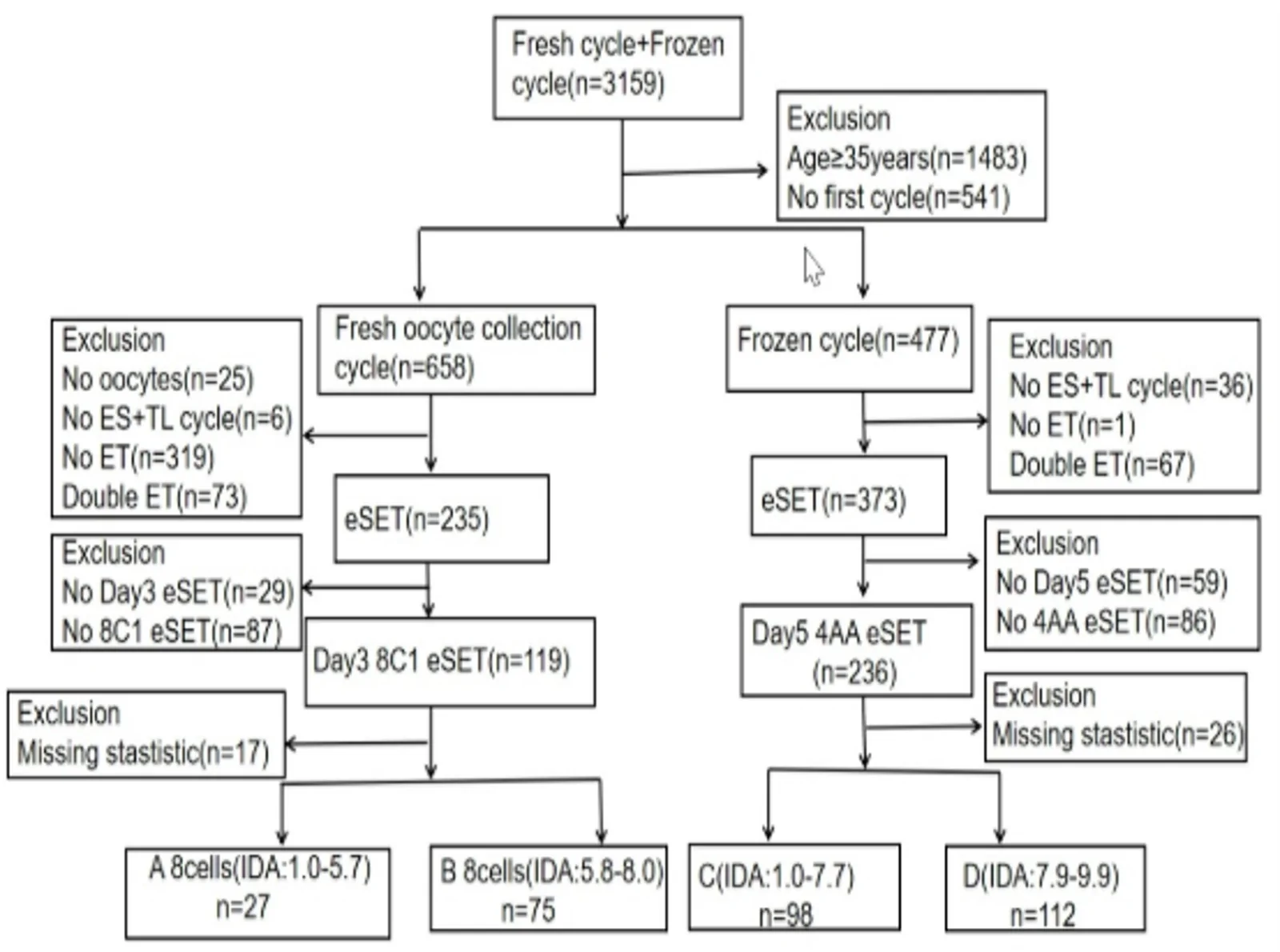
Flow chart of the study, eSET, elective single embryo transfer

Patients and Study Design: This retrospective study included patients who underwent IVF/ICSI treatment at the Reproductive Medicine Center of the Affiliated Hospital of Guangdong Medical University between August 2023 and March 2025. All procedures involving human participants were conducted in accordance with the ethical standards of the institutional and national research committees, as well as the 1964 Declaration of Helsinki and its subsequent amendments. All patients provided written informed consent and were informed of their right to withdraw from the study at any time. This study was approved by the Clinical Research Ethics Committee of the Affiliated Hospital of Guangdong Medical University (Approval No. PJKT2025-072). Inclusion Criteria:1.Patients undergoing IVF/ICSI using either the GnRH antagonist or long agonist protocols; 2.Female age < 35 years; 3.Embryos morphologically graded as 8-cell grade 1 (8C1) or blastocyst grade 4AA by manual assessment, and evaluated using the IDAScore model; 4.Transfer of a single Day 3 embryo in a fresh cycle or the first frozen-thawed single Day 5 blastocyst transfer cycle; 5.Complete cycle data available. Exclusion Criteria:1.Incomplete cycle data; 2.Female reproductive system abnormalities or chromosomal abnormalities in either partner; 3.Presence of conditions severely affecting IVF/ICSI outcomes, such as diminished ovarian reserve, endometrial disease, adenomyosis, or other uterine cavity disorders. The 8C1 cleavage embryos and 4AA blastocysts incorporated into this study were all obtained from retrieved MII mature oocytes, with exclusion of any embryos derived from immature oocytes. After applying the inclusion and exclusion criteria, a total of 102 fresh transfer cycles involving 8C1 cleavage-stage embryos were included in this study. Based on the median IDAScore V2.0, patients were divided into two groups: Group A [8cells, IDA:1.0–5.7]*n* = 27and Group B [8cells, IDA:5.8–8.0]*n* = 75. Additionally, 210 frozen-thawed blastocyst transfer cycles were analyzed and classified into two groups according to the median IDAScore: Group C [IDA:1.0–7.7]*n* = 98 and Group D [IDA:7.8–9.9]*n* = 112(Fig. 1). Comparisons were made among these four groups.

### Ovarian stimulation and oocyte retrieval

Ovarian stimulation was performed using either a GnRH antagonist protocol or a long GnRH agonist protocol, depending on the patient’s ovarian response and previous ART history. When at least one follicle reached 19 mm in diameter, or two follicles reached 18 mm, or three follicles reached 17 mm, and serum estradiol (E₂) levels were approximately 250–300 pg/mL per dominant follicle (≥ 16 mm), or more than 60% of follicles were ≥ 16 mm in diameter, human chorionic gonadotropin (HCG) (5,000–10,000 IU) was administered that evening to trigger final oocyte maturation. Transvaginal ultrasound-guided oocyte retrieval was performed 34–36 h later, followed by routine luteal phase support.

### Embryo culture and transfer

Depending on the patient’s condition, in vitro fertilization (IVF) or intracytoplasmic sperm injection (ICSI) was performed. After fertilization, all embryos were transferred into G-TL culture medium (Vitrolife, Sweden) and cultured in an FDA-approved time-lapse incubator (EmbryoScope Plus, Vitrolife, Denmark). EmbryoViewer software version 7.8.2 (Vitrolife, A/S, Denmark) was used for image acquisition and analysis. Embryos were cultured at 37 °C under conditions of 6% CO₂ and 5% O₂, with images captured every 10 min continuously over six days. For fresh embryo transfer, a single Day 3 cleavage-stage embryo (8C1) was selected for transfer. For frozen-thawed cycles, a single Day 5 blastocyst (graded 4AA) was transferred after endometrial preparation.

### Time-lapse imaging system and embryo assessment

EmbryoViewer software was used to collect data on 2PN embryos and to analyze time-lapse images throughout embryo development. The system allowed for retrospective review of embryo morphology and the documentation of key morphokinetic parameters, Including the times for pronuclear disappearance, 2-cell, 3-cell, 4-cell, 5-cell, 8-cell stages, and the time at which the blastocoel fills the entire zona pellucida (tPNf, t2, t3, t4, t5, t8, tB), representing the interval times between the 2nd and 3rd cell divisions, cc2 (t3 - t2) and cc3 (t5 - t3), and the synchronization indicators for the 2nd and 3rd cell divisions, s2 (t4 - t3) and s3 (t8 - t5).During the entire embryo culture process, IDAScore—according to the manufacturer’s settings—utilizes the built-in imaging system of the ESP incubator to automatically capture images of embryos every 10 min, generating continuous time-lapse videos and enabling real-time, second-level scoring. Embryo morphology was assessed based on the Istanbul consensus criteria [22]and the Gardner blastocyst grading system [23].

### Assessment of clinical outcomes

A serum hCG level > 5 U/L measured 14 days after embryo transfer was considered a positive biochemical pregnancy. Transvaginal ultrasound was performed 28 days after transfer to confirm intrauterine gestation. The presence of early fetal cardiac activity was defined as a clinical pregnancy.

### Outcome measures

The following parameters were collected and analyzed: Baseline patient characteristics: age, body mass index (BMI), baseline hormone levels, etc.; Ovarian stimulation data: the total dose of gonadotropins (Gn), hormone levels at trigger day, etc.; Embryo morphokinetic parameters: the time of pronuclear fading (tPNf), time to 3-cell stage (t3), 4-cell stage (t4), 5-cell stage (t5), 8-cell stage (t8), synchrony of the second cleavage (s2), duration from 2-cell to 3-cell stage (cc2), and time of full blastocyst expansion (tB), etc.; Embryo transfer outcomes: high-quality embryo rate(high-quality embryo/normally fertilized zygotes), clinical pregnancy rate(clinical pregnancies/transfer cycles), miscarriage rate(miscarriages / clinical pregnancies), blastocyst formation rate(blastocysts/blastocyst-cultured embryos), etc. All embryonic morphokinetic parameters and IDAScore scores analyzed in the present study were exclusively derived from single transferred embryos. Morphokinetic variable quantification was not conducted for the other untransferred embryos retrieved from the same treatment cycle; these spare embryos were only applied to comparative analyses of aggregate developmental indicators, such as the high-quality embryo rate and blastocyst formation rate.

### Statistical analysis

Data were analyzed using SPSS version 26.0 (IBM Corp., Armonk, NY, USA). Continuous variables with a normal distribution were expressed as mean ± standard deviation (x̄ ± s) and compared between groups using independent-samples t-tests. Skewed data were presented as median (M) and interquartile range (IQR), and compared using non-parametric tests. Categorical variables were presented as frequency and percentage [n (%)] and compared using the chi-square (χ²) test. Univariate and multivariate logistic regression analyses were performed to evaluate the relative importance of potential predictive variables in explaining IDAScore ratings. A p-value < 0.05 was considered statistically significant.

## Results

Comparison of baseline characteristics between patients in Group A and Group B undergoing cleavage-stage embryo transfer: After applying the inclusion and exclusion criteria, a total of 102 fresh single cleavage-stage embryo transfer cycles were included in the analysis. There were no statistically significant differences between Group A and Group B in terms of baseline characteristics, including age, body mass index (BMI), anti-Müllerian hormone (AMH) levels, duration of infertility, and baseline hormone levels (all *P* > 0.05) (Table 1).

**Table 1:** Comparison of General Characteristics Between Groups A and B [(X̄ ± s), M (P25, P75)]

Group	Group(A)[8cells(IDA:1.0-5.7)]	Group(B)[8cells(IDA:5.8-8.0)]	*p*-value
Cases	27	75	
Age	31.00(28.00,32.00)	30.00(28.00,32.00)	0.930
Duration of infertility	3.00(2.00,5.00)	3.00(1.00,5.00)	0.401
BMI(kg/m^2^)	21.81(19.33,23.62)	22.15(20.70,24.46)	0.483
bFSH(IU/L)	6.35(5.50,7.19)	6.42(5.33,7.09)	0.970
bE2(pg/ml)	52.10(31.80,59.80)	39.39(30.40,50.90)	0.142
bLH(IU/L)	5.41(4.24,7.75)	5.38(4.12,7.14)	0.685
bT(ng/ml)	0.33 ± 0.17	0.30 ± 0.11	0.538
bPRL(ng/ml)	21.00(15.12,32.50)	19.17(13.20,28.90)	0.413
bP(ng/ml)	0.35(0.13,0.45)	0.38(0.24,0.52)	0.165
AMH(ng/ml)	3.29(2.11,4.10)	2.85(1.78,4.50)	0.603
AFC, num	20.00(10.00,27.00)	18.00(12.00,29.00)	0.724
Fertilization method			0.203
IVF(%)	81.5(22/27)	70.7(53/75)	
ICSI(%)	18.5(5/27)	29.3(22/75)	

Comparison of Ovarian Stimulation Cycles Between Groups A and B: There were no significant differences between the two groups in terms of ovarian stimulation protocols, initial gonadotropin (Gn) dose, total Gn dose, Length of Gn stimulation, hormone levels at trigger day, or endometrial thickness at the trigger day (all *P* > 0.05) (Table 2).

**Table 2:** Comparison of Ovarian Stimulation Cycles Between Groups A and B [(X̄ ± s), M (P25, P75), %]

Group		Group(A)[8cells(IDA:1.0-5.7)]	Group(B)[8cells(IDA:5.8-8.0)]	*p*-value
No.of cycles		27	75	
Ovulation programme(%)	Long agonist protocols	33.3(9/27)	45.3(34/75)	0.279
	Antagonist protocol	66.7(18/27)	54.7(41/75)	
Initial Gn dose(IU)		150.00(112.50, 225.00)	150.00(137.50, 200.00)	0.682
No.of cycles		27	75	
Total dose of Gn(IU)		1800.00(1475.00, 2550.00)	1800.00(1500.00, 2400.00)	0.864
Length of Gn stimulation(days)		10.00(10.00,12.00)	10.00(9.00,11.00)	0.696
P at trigger day (ng/ml)		0.66 ± 0.28	0.71 ± 0.32	0.480
LH at trigger day (IU/L)		2.52(1.87,3.11)	2.77(1.78,3.94)	0.335
E2 at trigger day(ng/L)		2609.00(1919.00, 2991.00)	2464.00(1843.00, 2819.00)	0.764
Endometrial thickness on trigger day(mm)		11.00(10.00,14.00)	13.00(10.00,14.00)	0.491

Comparison of morphokinetic parameters between between groups A and B: There were no significant differences between Group A and Group B in tPNF, t2, s2, or s3 (*P* > 0.05). However, the IDAScore was significantly higher in Group B than in Group A (*P* < 0.05). Moreover, the time to reach the 3-cell(t3), 4-cell(t4), 5-cell(t5), and 8-cell stages(t8), as well as the durations from the 2-cell to the 3-cell stage(cc2) and from the 3-cell to the 5-cell stage(cc3), were significantly shorter in Group B compared to Group A (*P* < 0.05) (Table 3).

**Table 3:** Comparison of Morphokinetic Parameters Between Group A and Group B[(X̄ ± s), M(P25,P75)]

Group	Group(A)[8cells(IDA:1.0-5.7)]	Group(B)[8cells(IDA:5.8-8.0)]	*p*-value
No.of cycles	27	75	
IDAScore	5.00(4.40,5.50)	6.70(6.40,7.00)	<0.001
tPNF	23.54 ± 2.65	22.93 ± 1.96	0.211
t2	26.00 ± 2.64	25.37 ± 1.91	0.189
t3	37.93 ± 3.50	36.35 ± 2.31	0.036
t4	38.59 ± 3.75	36.95 ± 2.34	0.040
t5	52.97 ± 4.96	49.92 ± 3.44	0.006
t8	58.61 ± 5.22	55.25 ± 4.78	0.003
s2(t4-t3)	0.30(0.20,0.70)	0.40(0.20,0.70)	0.547
s3(t8-t5)	3.70(3.00,7.20)	4.40(2.10,7.00)	0.635
cc2(t3-t2)	11.93 ± 1.27	10.98 ± 0.88	0.001
No.of cycles	27	75	
cc3(t5-t3)	14.90(12.90,17.20)	13.60(12.50,14.90)	0.015

Comparison of Pregnancy Outcomes Between Group A and Group B: Group B demonstrated significantly higher rates of high-quality embryos, blastocyst formation, usable blastocysts, and clinical pregnancy compared to Group A (*P* < 0.05) (Table 4).

**Table 4:** Comparison of Clinical Outcome Indicators Between Group A and Group B[(X̄ ± s), M (P25, P75), %]

Group	Group(A)[8cells(IDA:1.0-5.7)]	Group(B)[8cells(IDA:5.8-8.0)]	*p*-value
No.of cycles	27	75	
Number of oocytes retrieved(n)	13.00(10.00,18.00)	11.00(10.00,14.00)	0.581
Total cleaved embryos	8.00(6.00,10.00)	8.00(7.00,11.00)	0.477
4-cell rate for Day2(%)	53.75 ± 25.10	62.16 ± 21.09	0.094
8-cell rate for Day3(%)	50.00(33.33,57.14)	50.00(37.50,66.67)	0.154
Rate of high-quality embryos(%)	33.33(27.27,53.33)	50.00(40.00,71.43)	0.003
Rate of blastocyst formation(%)	50.00(20.00,66.67)	71.43(45.45,85.71)	0.006
Rate of usable blastocysts(%)	60.00(0.00,100.00)	90.00(75.00,100.00)	<0.001
Clinical pregnancy (%)	33.3(9/27)	57.3(43/75)	0.032
Miscarriage rate (%)	0(0/9)	9.3(4/43)	1.000

Univariate and multivariate logistic regression analysis of morphokinetic parameters associated with D3 embryo IDAScore: Univariate analysis revealed that t3, t4, t5, t8, cc2, and cc3 were all significantly associated with D3 embryo IDAScore. Further multivariate logistic regression using a forward stepwise approach identified cc2 as an independent factor influencing D3 embryo score (Table 5).

**Table 5:** Logistic Regression Analysis of Morphokinetic Parameters Influencing D3 Embryo IDAScore

Morphokinetic Parameters	OR(95%CI)	Univariate analysis *p* value	OR(95%CI)	Multivariate analysis *p* value
t3	0.804(0.676,0.957)	0.014		
t4	0.812(0.689,0.958)	0.013		
t5	0.810(0.709,0.925)	0.002		
t8	0.875(0.797,0.960)	0.005		
cc2	0.410(0.253,0.666)	<0.001	0.410(0.253,0.666)	<0.001
cc3	0.716(0.565,0.907)	0.006		

Comparison of Baseline Characteristics Between Group C and Group D (Blastocyst Transfer Patients): After applying the inclusion and exclusion criteria, a total of 210 first frozen–thawed embryo transfer (FET) cycles were included in the analysis. There were no significant differences between the two groups in terms of age, body mass index (BMI), anti-Müllerian hormone (AMH) levels, duration of infertility, or baseline hormone levels (*P* > 0.05) (Table 6).

**Table 6:** Comparison of Baseline Characteristics Between Group C and Group D[(X̄ ± s), M(P25,P75),%]

Group	Group(C)(IDA:1.0-7.7)	Group(D) (IDA:7.8–9.9)	*p*-value
No.of cycles	98	112	
Female age(years)	30.50(29.00,32.00)	30.50(28.00,33.00)	0.882
Infertility duration(years)	2.35(1.00,4.00)	2.00(1.00,4.00)	0.407
BMI(kg/m2)	22.16 ± 2.86	21.93 ± 3.27	0.589
bFSH(IU/L)	6.29(5.37,7.21)	5.91(5.24,6.81)	0.119
bE2(pg/ml)	43.60(32.58,59.43)	40.50(30.01,57.70)	0.257
bLH(IU/L)	6.26(4.59,8.48)	6.57(4.71,8.87)	0.660
AMH(ng/ml)	4.09(2.50,6.23)	4.76(3.09,6.64)	0.101
Type of infertility			0.644
Primary infertility(%)	54.1(53/98)	50.9(57/112)	
Secondary infertility(%)	45.9(45/98)	49.1(55/112)	
Ovulation programme			0.140
Stimulated cycle(%)	8.2(8/98)	4.5(5/112)	
Hormone replacement therapy (HRT) cycle(%)	80.6(79/98)	90.2(101/112)	
No.of cycles	98	112	
Natural cycle(%)	11.2(11/98)	5.4(6/112)	
Fertilization method			0.723
IVF(%)	76.5(75/98)	78.6(88/112)	
ICSI(%)	23.5(23/98)	21.4(24/112)	

Comparison of Morphokinetic Parameters Between Groups C and D: The IDAScore was significantly higher in Group D than in Group C (*P* < 0.05). The time to reach the 2-cell (t2), 3-cell (t3), 4-cell (t4), 5-cell (t5) stages, and the time of full blastocyst expansion (tB) were all significantly shorter in Group D compared to Group C (*P* < 0.05). In addition, there were significant differences between the two groups in inner cell mass (ICM) grade and trophectoderm (TE) grade (*P* < 0.05) (Table 7).

**Table 7:** Comparison of Morphokinetic Parameters Between Group C and Group D[(X̄ ± s), M(P25,P7 ),%]

Group	Group(C)(IDA:1.0-7.7)	Group(D) (IDA:7.8–9.9)	*p*-value
No.of cycles	98	112	
IDAScore	6.90(6.20,7.33)	8.30(8.10,8.60)	<0.001
t2	25.20(23.75,26.80)	24.35(23.10,25.90)	0.022
t3	36.28 ± 3.21	35.28 ± 2.80	0.018
t4	37.00(34.70,38.63)	35.85(33.90,37.50)	0.010
t5	49.80(46.60,53.00)	48.40(45.93,50.65)	0.021
cc2(t3-t2)	11.00(10.28,11.80)	10.80(10.20,11.40)	0.100
s2(t4-t3)	0.40(0.20,0.90)	0.40(0.20,0.70)	0.387
cc3(t5-t3)	13.55(12.18,15.00)	13.25(11.90,14.20)	0.053
tB	103.52 ± 5.00	98.07 ± 4.29	<0.001
Morphological grade of inner cell mass			0.003
A(%)	77.6(76/98)	92.0(103/112)	
B(%)	22.4(22/98)	8.0(9/112)	
Morphological grade of trophectoderm			<0.001
A(%)	69.4(68/98)	92.0(103/112)	
B(%)	30.6(30/98)	8.0(9/112)	

Comparison of Pregnancy Outcomes Between Groups C and D: Group D showed a significantly higher blastocyst formation rate and a lower miscarriage rate compared to Group C, with both differences being statistically significant (*P* < 0.05) (Table 8).

**Table 8:** Comparison of Clinical Outcome Indicators Between Group C and Group D[( X ®±s), M(P25,P75 ),%]

Group	Group(C)(IDA:1.0-7.7)	Group(D) (IDA:7.8–9.9)	*p*-value
No.of cycles	98	112	
Number of oocytes retrieved	19.00(13.00,25.25)	19.00(14.00,24.00)	0.830
Normal Fertilization Rate (%)	89.47(81.82,96.19)	90.00(78.67,100.00)	0.984
Normal cleavage rate(%)	90.45(82.95,100.00)	90.24(79.00,100.00)	0.899
4-cell rate for Day2(%)	58.71(41.67,70.65)	60.77(46.67,74.67)	0.102
8-cell rate for Day3(%)	43.63 ± 19.00	47.31 ± 20.27	0.178
Rate of high-quality embryos(%)	44.10(33.33,64.39)	50.00(37.74,63.91)	0.051
Rate of blastocyst formation (%)	67.32 ± 18.05	73.50 ± 16.64	0.011
Rate of usable blastocysts(%)	83.33(75.00,100.00)	83.33(75.00,100.00)	0.982
Biochemical pregnancy rate(%)	73.5(72/98)	77.7(87/112)	0.478
Clinical pregnancy (%)	66.3(65/98)	74.1(83/112)	0.349
Miscarriage rate(%)	16.9(11/65)	6.0(5/83)	0.034

Univariate and Multivariate Logistic Regression Analysis of Morphokinetic Parameters Associated with Blastocyst IDAScore: Univariate analysis revealed that t2, t3, t4, t5, tB, ICM grade, and TE grade were all significantly associated with blastocyst IDAScore. Further multivariate logistic regression using a forward stepwise approach showed that tB and TE grade remained as significant factors influencing blastocyst scoring (Table 9).

**Table 9:** Logistic Regression Analysis of Morphokinetic Parameters Influencing Blastocyst IDAScore

Morphokinetic Parameters	Group	OR(95%CI)	Univariate analysis *p* value	OR(95%CI)	Multivariate analysis *p* value
t2		0.835(0.735,0.948)	0.005		
t3		0.894(0.813,0.982)	0.020		
t4		0.902(0.824,0.988)	0.027		
t5		0.930(0.877,0.987)	0.017		
tB		0.779(0.722,0.840)	<0.001	0.780(0.721,0.845)	<0.001
Morphological grade of inner cell mass	A	3.313(1.444,7.599)	0.005		
	B				
Morphological grade of trophectoderm	A	5.049(2.256,11.298)	<0.001	4.224(1.633, 10.926)	0.003
	B				

## Discussion

Accurate assessment and selection of high-quality embryos for elective single embryo transfer (eSET) has emerged as a key focus in reproductive medicine. Both fresh and frozen eSET cycles have demonstrated live birth rates comparable to those of double embryo transfer (DET), while significantly reducing the incidence of multiple gestations [24, 25]. Both cleavage-stage embryo transfer and blastocyst transfer offer distinct advantages. Previous studies have shown that blastocyst transfer effectively eliminates embryos with limited developmental potential, enhances implantation rates, and circumvents the vulnerability of cleavage-stage embryos to cryodamage. This approach also increases the likelihood of selecting the most viable embryo for transfer, making it the preferred strategy in most assisted reproduction centers worldwide [26, 27]. Fresh single cleavage-stage embryo transfer offers distinct advantages, including a reduced in vitro culture duration and shorter waiting time for embryo implantation. Moreover, it lowers the risk of cycle cancellation due to blastocyst culture failure, thereby providing a pragmatic and effective alternative in selected cases [28, 29].

Embryo developmental potential is a critical determinant in the selection of single embryos for transfer and is closely associated with clinical outcomes in assisted reproductive technology (ART). The TL system, as a novel non-invasive embryo culture and evaluation platform, minimizes the need for repeated embryo handling and removal from the incubator, thereby maintaining a more stable and secure environment for embryo development [30, 31]. Since its implementation, time-lapse TL imaging has enabled the identification of key morphokinetic parameters predictive of embryo development and clinical outcomes. In a large multicenter, single-blind, randomized controlled trial, embryos from 1,182 participants undergoing embryo transfer were randomly assigned in a 1:1 ratio to either the TL group or the conventional incubation group. The results showed that the number of successfully implanted embryos in the TL group during the first embryo transfer cycle was significantly higher [32]. Kalleas et al. [33] reported that patients in the TL culture group had a significantly lower early miscarriage rate and a significantly higher live birth rate.

Relevant morphokinetic parameters were shown to predict embryo quality, blastocyst formation rate, embryo survival rate, implantation rate, pregnancy outcomes, live birth rate, and even the potential to assess embryo ploidy [34, 35]. Our findings demonstrate that significant differences were observed in the early cleavage time points—t3, t4, t5, t8, cc2, and cc3—between the two groups of cleavage-stage embryos. Similarly, in the blastocyst-stage embryos, the timing of key developmental milestones including t2, t3, t4, t5, and full blastocyst expansion (tB) differed significantly between the groups. Notably, comparisons of pregnancy outcomes across these groups also revealed statistically significant differences. Cleavage-stage embryos exhibiting faster developmental kinetics demonstrated higher rates of high-quality embryos, blastocyst formation, usable blastocysts, and clinical pregnancy compared to those with slower cleavage. Similarly, blastocysts derived from rapidly dividing embryos had a lower miscarriage rate and a higher blastocyst formation rate than those with delayed development.

Certain parameters in this study are consistent with the findings reported by Meseguer et al. [36], who identified t5 and cc2 as being significantly associated with implantation rates. Similarly, Sayed et al. [37]supported this conclusion, highlighting t5 and cc2 as the most predictive markers for successful implantation and live birth outcomes. Bori et al. [12]demonstrated that t2, t3, t4, t5, and tB were significantly associated with embryo implantation potential. Similarly, Goodman et al. [38] identified t5, tSB, cc2, s2, and s3 as predictive parameters for blastocyst formation. Milewski et al. [39]also found a strong correlation between t2, t5, cc2 and the likelihood of blastocyst development. Furthermore, Hashimoto et al. [40]analyzed the timing of t3 and t4 divisions, concluding that high-grade blastocysts reached these developmental milestones significantly earlier than their lower-quality counterparts. Coticchio et al. [41] observed that accelerated fertilization kinetics are associated with improved embryonic development, which is consistent with the findings of our study. Variations in the morphokinetic parameters used to predict embryonic outcomes across different studies may be attributed to differences in culture environments and conditions, which can significantly influence embryo growth and developmental dynamics.

TL technology has been increasingly adopted in clinical practice, resulting in the accumulation of extensive standardized embryo image and video datasets. This has garnered growing interest among embryologists in leveraging artificial intelligence (AI) in conjunction with TL systems to rank and select embryos for transfer or cryopreservation. The integration of TL technology with artificial intelligence (AI) allows for the analysis, recognition, classification, automatic annotation, and evaluation of embryo development videos [17], serving as the primary foundation for AI-based embryo selection. By incorporating large-scale datasets and enabling autonomous learning, these AI-driven models can be further optimized to more accurately predict embryo implantation potential, blastocyst formation rate, and pregnancy success rate, thereby enhancing the overall efficiency of assisted reproductive technology (ART) treatments [42–44].

Many AI-based embryo selection models are developed using deep learning algorithms. Jørgen Berntsen et al. [45]created a fully automated model, IDAScore v1.0, by analyzing a large TL technology dataset collected from 18 IVF centers. The scores generated by this model were positively correlated with embryo quality. In a recent study [46], IDAScore v2.0 demonstrated significantly higher accuracy in predicting pregnancy outcomes across all age cohorts compared to both IDAScore v1.0 and the traditional Gardner grading system. Similarly, findings by Ahlstrom A et al. [47]indicated that TL imaging combined with the IDAScore model could also predict the developmental potential of individual embryos. Papamentzelopoulou MS et al. [48]reported that blastocysts with higher KIDScore and iDAScore values were associated with increased live birth rates.In this study, we found that although all embryos were classified as 8C1 embryos based on conventional morphological criteria, those with higher time-lapse–derived scores at the cleavage stage exhibited significantly higher rates of high-quality embryos, blastocyst formation, usable blastocysts, and clinical pregnancy. Moreover, high-scoring blastocysts demonstrated a significantly higher blastocyst formation rate and a lower miscarriage rate compared to their low-scoring counterparts. In a previous study from our center [49], which also showed that, among embryos with identical morphological grades, the high-score group had a greater number of high-quality embryos, more blastocysts formed, and a higher blastocyst formation rate than the low-score group. These provides important reference for selecting high-scoring embryos for transfer in patients with multiple high-quality embryos, thereby improving the clinical outcomes of embryo transfer. However, the lack of transparency in the internal decision-making processes of AI models remains a key limitation to their broader application in the medical field. To improve algorithmic accuracy, AI models often rely on the analysis of direct cleavage events and the timing intervals between normal cell divisions [45]. One potential approach to enhancing model interpretability is to evaluate the correlation between the outputs of AI-based embryo selection models and traditional morphokinetic parameters.

The results of this study revealed that the IDAScore was significantly correlated with cleavage-stage morphokinetic parameters (t3, t4, t5, t8, cc2, cc3) and blastocyst development parameters (t2, t3, t4, t5, tB), as well as with traditional morphological assessments including inner cell mass (ICM) and trophectoderm (TE) grading. Logistic regression analysis further indicated that cc2 remained an independent predictor of Day 3 embryo scores, while tB and TE grading were significant predictors of blastocyst scores.In this study, all blastocysts received an A grade for both ICM and TE under conventional static morphological assessment. However, some of these same blastocysts were graded as B by the time-lapse (TL) system. This discrepancy may stem from the limitations of traditional morphology, which relies on static images, brief observation periods, and high subjectivity. In contrast, the TL system integrates dynamic developmental information, continuous monitoring, and environmental factors, providing a more comprehensive evaluation of embryo quality. Ezoe et al. [20]demonstrated that IDAScore outputs are associated with preimplantation embryo morphokinetics and morphological changes. Embryos with higher IDAScore values reached developmental milestones more rapidly than those with lower scores, a finding consistent with the results of our study. This observation is further supported by the IDAScore model developed by Jørgen Berntsen [46], which showed that IDAScore is closely linked to embryo developmental kinetics and morphological parameters. Specifically, the model assigns lower scores to embryos exhibiting direct cleavage, while favoring faster-developing blastocysts and those with high-quality trophectoderm (TE) and inner cell mass (ICM), despite not being explicitly trained on these individual features. This suggests that the model implicitly recognizes the predictive importance of these parameters for implantation potential.

In summary, outputs generated by the IDAScore embryo screening model are significantly correlated with embryonic morphokinetic parameters and pregnancy outcomes. The findings of this study further provide evidence for the independent morphokinetic markers underlying IDAScore values and verify that the model maintains stable discriminatory performance in Asian populations. This work facilitates clinicians, embryologists and patients to better comprehend and trust its evaluation results, relieving doubts regarding the accuracy of this AI model when applied to Asian populations and improving its generalizability for use in reproductive centers worldwide. The relatively small single-center sample size and exclusive focus on embryos transferred in the first treatment cycle may introduce bias into our results. Further validation based on high-quality datasets and large-sample multicenter studies is therefore warranted. In addition, this study did not conduct subgroup stratified analyses according to male or female infertility etiologies, nor did we explore differences in ovarian stimulation protocols among patients receiving blastocyst transfers. Large-scale multicenter investigations with high-quality data are needed to externally validate our outcomes. Subgroup analyses based on male or female infertility factors were not carried out herein. Future multicenter studies with expanded sample sizes may stratify patients by infertility origin to clarify potential modifying impacts of infertility etiology on iDAScore-morphokinetic associations and yield more definitive conclusions. When applying iDAScore clinically, raw embryo scores should undergo center-specific calibration adjusting for ovarian function confounders such as maternal age, AMH and AFC to correct score discrepancies related to diverse age and ovarian reserve statuses and optimize grading accuracy for local populations. Moreover, ethics design and patient privacy risks associated with AI implementation should be fully addressed.

## Data Availability

No datasets were generated or analysed during the current study.
